# β-Adrenergic blockade in patients with dementia and hip fracture is associated with decreased postoperative mortality

**DOI:** 10.1007/s00068-021-01723-y

**Published:** 2021-06-15

**Authors:** Ioannis Ioannidis, Ahmad Mohammad Ismail, Maximilian Peter Forssten, Yang Cao, Gary Alan Bass, Tomas Borg, Shahin Mohseni

**Affiliations:** 1 Department of Orthopedic Surgery, Orebro University Hospital, 701 85 Orebro, Sweden; 2 School of Medical Sciences, Orebro University, 702 81 Orebro, Sweden; 3 Clinical Epidemiology and Biostatistics, School of Medical Sciences, Orebro University, 701 82 Orebro, Sweden; 4 Division of Traumatology, Surgical Critical Care and Emergency Surgery, University of Pennsylvania, Philadelphia, USA; 5 Division of Trauma and Emergency Surgery, Department of Surgery, Orebro University Hospital, 701 85 Orebro, Sweden

**Keywords:** Hip fracture, Dementia, Beta-blocker, β-Adrenergic blockade, Mortality

## Abstract

**Purpose:**

Dementia, present in 20% of hip fracture patients, is associated with an almost threefold increase in postoperative mortality risk. These patients have a substantially higher incidence of cardiovascular, respiratory, and cerebrovascular mortality after hip fracture surgery compared to patients without dementia. This study aimed to investigate the association between beta-blocker therapy and postoperative mortality in patients with dementia undergoing hip fracture surgery.

**Methods:**

This nationwide study included all patients in Sweden with the diagnosis of dementia who underwent emergency surgery for a hip fracture between January 2008 and December 2017. Cases where the hip fracture was pathological or conservatively managed were not included. Poisson regression analysis with robust standard errors was performed while controlling for confounders to determine the relationship between beta-blocker therapy and all-cause, as well as cause-specific, postoperative mortality.

**Results:**

A total of 26,549 patients met the study inclusion criteria, of whom 8258 (31%) had ongoing beta-blocker therapy at time of admission. After adjusting for clinically relevant variables, the incidence of postoperative mortality in patients receiving beta-blocker therapy was decreased by 50% at 30 days [adj. IRR (95% CI) 0.50 (0.45–0.54), *p* < 0.001] and 34% at 90 days [adj. IRR (95% CI) 0.66 (0.62–0.70), *p* < 0.001]. Cause-specific mortality analysis demonstrated a significant reduction in the incidence of postoperative cardiovascular, respiratory, and cerebrovascular death within 30 and 90 days postoperatively.

**Conclusion:**

Beta-blocker therapy is associated with decreased postoperative mortality in hip fracture patients with dementia up to 90 days after surgery. This finding warrants further investigation.

**Supplementary Information:**

The online version contains supplementary material available at 10.1007/s00068-021-01723-y.

## Background

Despite interventions and guidelines introduced during the past decade to reduce mortality, postoperative mortality has remained a constant challenge in managing hip fracture patients [1]. Furthermore, approximately 20% of hip fracture patients have dementia, a condition independently associated with an almost threefold increase in postoperative mortality risk [2–6]. These patients have a substantially higher incidence of cardiovascular, respiratory, and cerebrovascular mortality after hip fracture surgery compared to patients without dementia [7]. The hyper-adrenergic state that follows a traumatic injury and subsequent surgery plays a role in these adverse outcomes [2, 8–12]. Beta-adrenergic blockade may reduce this hyper-adrenergic state and mitigate its toxic effect [11–14], in turn reducing mortality after traumatic injuries [2, 8, 9, 15]. Several previous studies have demonstrated a positive association between beta-blocker therapy (BB) and a reduction in postoperative mortality after non-cardiac surgery, including in patients with isolated hip fractures [2, 9, 10, 16]. This study investigates the association between beta-blocker therapy and postoperative mortality in patients with dementia undergoing hip fracture surgery, with the hypothesis that beta-blocker therapy is associated with decreased mortality.

## Methods

The Swedish National Ethical Review Authority approved the current study (reference 2020-04161), which adhered to the Declaration of Helsinki principles and the STROBE guidelines [17]. Rikshöft, the prospective Swedish national hip fracture quality registry, was used as the basis for the current study [18]. The date of hospital admission, age, sex, fracture type, American Society of Anesthesiologist (ASA) classification, surgical method, date of surgery, and hospital discharge date were retrieved from Rikshöft. Patients’ social security numbers were then used to cross-reference the data from Rikshöft with the time of death and comorbidity data from the Swedish National Board of Health and Welfare quality registers. An age-adjusted Charlson Comorbidity Index (CCI) was calculated from the comorbidity data [19]. All patients diagnosed with dementia who underwent primary emergency surgery for a hip fracture between January 1, 2008, and December 31, 2017 were included in the current study. Cases where the hip fractures were pathological, i.e. non-traumatic, or conservatively managed, were not included.

### Beta-blocker use

We further cross-referenced the Rikshöft database with the Swedish National Board of Health and Welfare Prescribed Drug Registry, a population-based database containing all prescriptions issued in Sweden, to link beta-blocker prescriptions to individual hip fracture patients. Beta-blocker use was defined as patients who filled a prescription for any beta-blocker (ACT codes C07AA, C07AB, and C07AG) within 12 months before the date of surgery. We selected 12 months since beta-blockers are rarely discontinued once prescribed and are commonly issued on a long-term basis, with a single prescription spanning up to 12 months. Information regarding dose and generic drug names was also collected from this database. According to national guidelines set by the Swedish Society of Anesthesiology and Intensive Care, ongoing beta-blocker therapy should not be discontinued before surgery in the absence of absolute contraindications.

### Statistical analysis

The cases were divided into two cohorts: ongoing beta-blocker therapy (BB^+^) and no beta-blocker therapy (BB^−^). Patient demographics and clinical characteristics were compared between the cohorts. Categorical variables were reported with percentages, while continuous variables were reported as a mean and standard deviation (SD) or median and interquartile range (IQR). Pearson’s Chi-squared test and Fisher’s exact test were used to determine the statistical significance of differences between categorical variables. The Student’s *t* test was used for the normally distributed variable, age. Length of stay was not normally distributed; hence, the Mann–Whitney *U* test was applied. The primary outcome of interest was 30-day postoperative all-cause and cause-specific mortality. The secondary outcome of interest was 90-day postoperative all-cause and cause-specific mortality.

A Poisson regression model was employed to investigate the association between beta-blocker therapy and 30-day and 90-day postoperative mortality. Analyses were performed while adjusting for age, sex, time to surgery, ASA classification, fracture type, type of surgery, prior myocardial infarctions, prior cerebrovascular events, peripheral vascular disease, chronic obstructive pulmonary disease, congestive heart failure, connective tissue diseases, diabetes mellitus, liver disease, chronic kidney disease, as well as local tumors and metastatic carcinoma. The analyses were repeated with 30- and 90-day cause-specific mortalities. Results are reported as incidence rate ratios (IRR) with 95% confidence intervals (CI). Statistical significance was defined as a two-sided *p* value less than 0.05. Analyses were performed using the statistical programming language R (R Foundation for Statistical Computing, Vienna, Austria) [20].

## Results

A total of 26,549 (19.7%) patients with dementia, out of 134,915 cases operated for hip fractures, fulfilled the inclusion criteria. A larger proportion of the BB^+^ cohort were females (71.1% vs. 68.3%, *p* < 0.001). Cervical hip fractures were slightly more common among BB^−^ patients (52.2% vs. 49.6%, *p* < 0.001). Fixation with pins or screws with and without a sideplate were marginally less common among BB^+^ patients (40.4% vs. 42.8%, *p* < 0.001). There was no clinically significant difference in the age of patients in the cohorts. The BB^−^ cohort had fewer comorbidities (CCI ≥ 7: 23.7% vs 39.8%, *p* < 0.001) and was more fit for surgery (ASA ≥ 3: 67.9% vs. 78.7%, *p* < 0.001). Metoprolol was the most common beta-blocker prescribed (60.7%), followed by bisoprolol (18.2%) and atenolol (12.2%). Time to surgery was significantly shorter in the BB^−^ cohort (Time to surgery < 24 h: 68.2% vs 64.4%, *p* < 0.001) (Table 1). All comorbidities were less prevalent in the BB^−^ cohort apart from metastatic carcinoma, which was equally prevalent in both cohorts (Table 2).

**Table 1:** Patient demographics and clinical characteristics in patients with dementia undergoing hip fracture surgery, with and without beta-blocker therapy

		BB − *N* = 18,291	BB + *N* = 8258	*p* value
Age in years, mean [SD]		85 [7]	85 [7]	0.019
Sex, *n* (%)				< 0.001
Female		12,500 (68.3)	5869 (71.1)	
Male		5791 (31.7)	2389 (28.9)	
Type of beta-blocker, *n* (%)				N/A
Metoprolol		−	5013 (60.7%)	
Bisoprolol		−	1505 (18.2%)	
Atenolol		−	1009 (12.2%)	
Other		−	731 (8.9%)	
Time to surgery, *n* (%)				< 0.001
< 24 h		12,470 (68.2)	5319 (64.4)	
> 24 h		5821 (31.8)	2939 (35.6)	
ASA classification, *n* (%)				< 0.001
1		239 (1.3)	44 (0.5)	
2		5300 (29.0)	1534 (18.6)	
3		10,826 (59.2)	5492 (66.5)	
4		1567 (8.6)	1000 (12.1)	
5		27 (0.1)	11 (0.1)	
Missing		332 (1.8)	177 (2.1)	
Charlson Comorbidity Index, *n* (%)				< 0.001
≤ 4		2062 (11.3)	436 (5.3)	
5–6		11,889 (65.0)	4537 (54.9)	
≥ 7		4340 (23.7)	3285 (39.8)	
Fracture type, *n* (%)				< 0.001
Non−displaced cervical (garden 1–2)		2551 (13.9)	989 (12.0)	
Displaced cervical (garden 3–4)		7004 (38.3)	3104 (37.6)	
Basicervical		623 (3.4)	286 (3.5)	
Pertrochanteric (two fragments)		3360 (18.4)	1527 (18.5)	
Pertrochanteric (multiple fragments)		3513 (19.2)	1696 (20.5)	
Subtrochanteric		1235 (6.8)	653 (7.9)	
Missing		5 (0.0)	3 (0.0)	
Type of surgery, *n* (%)				< 0.001
Pins or screws		3305 (18.1)	1385 (16.8)	
Screws or pins with sideplate		4512 (24.7)	1949 (23.6)	
Intramedullary rod		4262 (23.3)	2211 (26.8)	
Hemiarthroplasty		5839 (31.9)	2532 (30.7)	
Total hip replacement		365 (2.0)	179 (2.2)	
Missing		8 (0.0)	2 (0.0)	

*BB* − no beta-blocker therapy; *BB* +  ongoing beta-blocker therapy; *SD* standard deviation; *ASA* American Society of Anesthesiologists

**Table 2:** Preoperative comorbidities in patients with dementia undergoing hip fracture surgery, with and without beta-blocker therapy

	BB − *N* = 18,291	BB + *N* = 8258	*p* value
Hypertension, *n *(%)	5624 (30.7)	4617 (55.9)	< 0.001
Arrhythmia, *n *(%)	2194 (12.0)	2955 (35.8)	< 0.001
Myocardial infarction, *n *(%)	653 (3.6)	907 (11.0)	< 0.001
Heart failure, *n *(%)	2116 (11.6)	2363 (28.6)	< 0.001
Peripheral vascular disease, *n *(%)	495 (2.7)	431 (5.2)	< 0.001
Cerebrovascular event, *n *(%)	3521 (19.2)	2250 (27.2)	< 0.001
Chronic obstructive pulmonary disease, *n *(%)	1684 (9.2)	988 (12.0)	< 0.001
Connective tissue disease, *n *(%)	654 (3.6)	391 (4.7)	< 0.001
Peptic ulcer disease, *n *(%)	565 (3.1)	342 (4.1)	< 0.001
Liver disease, *n *(%)	94 (0.5)	89 (1.1)	< 0.001
Diabetes Mellitus, *n *(%)	2126 (11.6)	1632 (19.8)	< 0.001
Hemiplegia, *n *(%)	256 (1.4)	206 (2.5)	< 0.001
Chronic kidney disease, *n *(%)	678 (3.7)	632 (7.7)	< 0.001
Local tumor, *n *(%)	1741 (9.5)	870 (10.5)	0.011
Metastatic carcinoma, *n *(%)	262 (1.4)	104 (1.3)	0.290

*BB* − no beta-blocker therapy; *BB* +  ongoing beta-blocker therapy

Both crude 30- and 90-day postoperative mortality was lower in the in the BB^+^ cohort (30-day: 9.3% vs 14.5%, *p* < 0.001; 90-day: 18.5% vs 24.0%, *p* < 0.001) (Table 3). The incidence of 30-day postoperative mortality due to cardiovascular (3.7% vs. 5.2%, *p* < 0.001), respiratory (1.3% vs. 2.7%, *p* < 0.001), cerebrovascular (0.2% vs. 0.5%, *p* = 0.005) causes, and multi-organ dysfunction syndrome (3.1% vs. 4.8%, *p* < 0.001) were significantly lower in the BB^+^ cohort (Table 4). There were no statistically significant differences in the incidence of death attributable to sepsis or unknown causes 30 days postoperatively. The incidence of cardiovascular, respiratory, cerebrovascular, and multi-organ dysfunction syndrome deaths remained significantly lower at 90 days postoperatively in the BB^+^ cohort (Table 4).

**Table 3:** Crude outcomes in patients with dementia undergoing hip fracture surgery, with and without beta-blocker therapy

	BB − *N* = 18,291	BB + *N* = 8258	*p* value
Hospital length of stay, days			< 0.001
Median [IQR]	6 [4, 10]	7 [5, 11]	
Missing	163 (0.9)	62 (0.8)	
30-day mortality, *n* (%)	2655 (14.5)	765 (9.3)	< 0.001
90-day mortality, *n* (%)	4384 (24.0)	1528 (18.5)	< 0.001

*BB* − no beta-blocker therapy; *BB* +  ongoing beta-blocker therapy; *IQR* interquartile range

**Table 4:** Cause of mortality in patients with dementia undergoing hip fracture surgery, with and without beta-blocker therapy

Cause of mortality	30-day mortality BB − *N* = 18,291	30-day mortality BB + *N* = 8258	*p* value	90-day mortality BB − *N* = 18,291	90-day mortality BB + *N* = 8258	*p* value
Cardiovascular	950 (5.2%)	306 (3.7%)	< 0.001	1443 (7.9%)	583 (7.1%)	0.020
Respiratory	502 (2.7%)	104 (1.3%)	< 0.001	683 (3.7%)	165 (2.0%)	< 0.001
Cerebrovascular	86 (0.5%)	19 (0.2%)	0.005	263 (1.4%)	54 (0.7%)	< 0.001
Sepsis	57 (0.3%)	14 (0.2%)	0.052	107 (0.6%)	35 (0.4%)	0.120
MODS	882 (4.8%)	260 (3.1%)	< 0.001	1,481 (8.1%)	536 (6.5%)	< 0.001
Unknown	162 (0.9%)	55 (0.7%)	0.077	382 (2.1%)	144 (1.7%)	0.069

*BB* − no beta-blocker therapy; *BB* +  ongoing beta-blocker therapy, *MODS* multi-organ dysfunction syndrome

After adjusting for age, sex, time to surgery, ASA classification, fracture type, type of surgery, prior myocardial infarctions, prior cerebrovascular events, peripheral vascular disease, chronic obstructive pulmonary disease, congestive heart failure, connective tissue diseases, diabetes mellitus, liver disease, chronic kidney disease, as well as local tumors and metastatic carcinoma, the incidence of postoperative mortality in patients receiving beta-blocker therapy was 50% lower after 30 days [adj. IRR (95% CI) 0.50 (0.45–0.54), *p* < 0.001] and 34% lower after 90 days [adj. IRR (95% CI) 0.66 (0.62–0.70), *p* < 0.001]. The 30-day incidence of death due to cardiovascular events [adj IRR (95% CI) 0.48 (0.41–0.56), *p* < 0.001], respiratory disease [adj IRR (95% CI) 0.35 (0.27–0.46), *p* < 0.001], cerebrovascular events [adj IRR (95% CI) 0.52 (0.27–0.98), *p* = 0.041], sepsis [adj IRR (95% CI) 0.44 (0.20–0.97), *p* = 0.041], and multi-organ dysfunction syndrome [adj IRR (95% CI) 0.56 (0.47–0.65), *p* < 0.001] was also reduced in BB^+^ patients. These associations remained unchanged when analyzing 90-day cause-specific mortality, apart from mortality due to sepsis 90 days postoperatively (Table 5).

**Table 5:** Incidence rate ratio for mortality in patients with dementia on beta-blocker therapy undergoing hip fracture surgery

Variable	30-day IRR (95% CI)	*p* value	90-day IRR (95% CI)	*p* value
All-cause mortality				
BB −	ref		ref	
BB +	0.50 (0.45–0.54)	< 0.001	0.66 (0.62–0.70)	< 0.001
Cause-specific mortality				
Cardiovascular	0.48 (0.41–0.56)	< 0.001	0.64 (0.57–0.72)	< 0.001
Respiratory	0.35 (0.27–0.46)	< 0.001	0.43 (0.35–0.54)	< 0.001
Cerebrovascular	0.52 (0.27–0.98)	0.041	0.51 (0.36–0.73)	< 0.001
Sepsis	0.44 (0.20–0.97)	0.041	0.68 (0.41–1.14)	0.142
MODS	0.56 (0.47–0.65)	< 0.001	0.73 (0.65–0.82)	< 0.001
Unknown	0.73 (0.49–1.07)	0.108	0.89 (0.71–1.11)	0.304

Poisson regression models with robust standard errors. Multiple imputations with chained equations were used to manage missing values. The model is adjusted for age, sex, time to surgery, ASA classification, fracture type, type of surgery, prior myocardial infarctions, prior cerebrovascular events, peripheral vascular disease, chronic obstructive pulmonary disease, congestive heart failure, connective tissue diseases, diabetes mellitus, liver disease, chronic kidney disease, as well as local tumors and metastatic carcinoma

*IRR* incidence rate ratio; *CI* confidence interval; *BB* − no beta-blocker therapy; *BB* +  ongoing beta-blocker therapy, *MODS* multi-organ dysfunction syndrome

## Discussion

The association between beta-blocker therapy and postoperative mortality in patients with dementia and hip fractures has not been previously described. Poisson regression analysis demonstrated an association between patients with ongoing beta-blocker therapy and a reduction in postoperative mortality after both 30 and 90 days. Postoperative mortality resulting from cardiovascular, pulmonary, and cerebrovascular events was also significantly reduced in patients with ongoing beta-blocker therapy.

Despite interventions and guidelines aimed at improving perioperative care after hip fracture surgery, postoperative mortality remains high [1]. The majority of these deaths are due to cardiovascular or other causes not attributable to the surgical procedure’s technical aspects. The aggregation of comorbidities and aging into overall frailty may exacerbate the physiological stress response induced by the trauma, surgical procedure, and postoperative rehabilitation [1, 8, 21–23]. It has previously been postulated that the posttraumatic hyper-adrenergic state can be reduced with beta-blockers, thereby potentially reducing the mortality rate among patients who have experienced physical trauma [2, 8–12]. This association has been found in several studies conducted on trauma patients, major non-cardiac surgical procedures, such as elective abdominal colorectal cancer resections, emergency laparotomy in geriatric patients, and more recently in hip fracture patients [2, 9, 15, 24]. Ahl and colleagues reviewed 134,915 hip fracture cases, demonstrating a 72% reduction in the incidence of 30-day all-cause postoperative mortality in patients with ongoing beta-blocker therapy [adj. IRR (95% CI) 0.28 (0.26–0.29), *p* < 0.001] [2]. In the current study population, which only contains hip fracture patients diagnosed with dementia, beta-blocker therapy was associated with a 50% reduction in 30-day postoperative mortality.

Approximately 20% of the hip fracture patients have dementia, which has been strongly linked to worse outcomes [2–6], with a significantly higher risk of cardiovascular, respiratory, and cerebrovascular mortality after hip fracture surgery compared to patients without dementia [7]. In the last 15 years, two meta-analyses and a Cochrane review have been published on beta-blocker therapy and non-cardiac surgery, outlining its cardioprotective effects [25–27]. Consequently, international guidelines from the American Heart Association, American College of Cardiology (AHA/ACC), and the European Society of Cardiology (ESC) warn against discontinuing regular beta-blockers before or following any surgery. Expert consensus further recommends beta-blocker initiation in intermediate to high-risk patients [≥ 2 clinical risk factors, ASA class ≥ 3, and over three factors from the Revised Cardiac Risk Index (RCRI)] [28, 29]. Many hip fracture patients with dementia fall in the high-risk patient category and should be considered for preoperative beta-blocker initiation. In the current study population, over 65% of beta-blocker naive patients had an ASA class ≥ 3, a CCI score over 5, and multiple risk factors from the RCRI. These patients could potentially have benefited from perioperative beta-blocker therapy.

Perioperative myocardial infarction is associated with a fivefold increase in mortality. Thirty-four percent of all deaths within 90 days of the operation followed significant cardiac events, making it the most common cause of death in our study. Its incidence (per 1000 surgeries) increases from 0.064 in low-risk patients to 15.8% among higher-risk patients (ASA Class ≥ 3, age ≥ 80 years, high-risk surgery) [30]. The current study population, which is drawn from a national patient population, is both older (with a mean age of 85) and often has an ASA classification ≥ 3; accordingly, they have a high risk for postoperative myocardial infarction. Despite their more advanced age, greater comorbidity burden (including cardiovascular comorbidities), and higher preoperative ASA classifications, the BB^+^ cohort had lower crude mortality rates both 30 and 90 days postoperatively. Furthermore, the adjusted 30- and 90-day postoperative cardiovascular mortality risk was reduced by 52% and 36%, respectively, in the BB^+^ cohort compared to the BB^−^ cohort. This finding makes beta-blockers, which are both readily available, cheap, and have a well-known side-effect profile, very appealing for use in clinical practice.

The retrospective nature of this study restricts the analyses to available variables, prohibiting conclusions about causality. One limitation was the coding of dementia as a single diagnosis in the dataset; the authors recognize that dementia describes a spectrum of conditions with a large degree of heterogeneity in their pathophysiology and mortality rates. The severity of dementia and preoperative functional status of each patient was also unknown. Regarding beta-blocker therapy, we used the prescription data to make reasonable assumptions about which patients were receiving ongoing beta-blocker therapy at the time of surgery. However, we could neither be confident of full compliance with treatment nor eliminate the risk that there was a discontinuation of therapy within that period. However, the authors believe discontinuation for such therapy to be highly unlikely since this is against the guidelines [28, 29]. Further, a previous study from our institution, including 2443 consecutive hip fracture cases, did not find a single case of beta-blocker therapy being discontinued [8].

The current study also demonstrates several strengths. The Rikshöft national database provides one of the largest sample populations to date studying postoperative mortality in hip fracture patients with dementia. Additionally, the universal healthcare system available in Sweden aids in the reduction of socioeconomic disparities in healthcare outcomes [31]. This is particularly important in patients with dementia since their condition inherently places them at a socioeconomic disadvantage [32].

## Conclusion

Beta-blocker therapy is associated with decreased postoperative mortality in hip fracture patients with dementia up to 90 days. This treatment could abrogate the high risk of postoperative mortality in this vulnerable patient population and should be further investigated, preferably in a pragmatic prospective randomized controlled trial.

## Supplementary Information

Below is the link to the electronic supplementary material.

Supplementary file1 (PDF 6159 KB)

## Data Availability

May be made available on reasonable request provided the appropriate ethical approval is sought and approved.
